# RNA *N^6^*-methyladenosine reader YTHDC1 is essential for TGF-beta-mediated metastasis of triple negative breast cancer

**DOI:** 10.7150/thno.71872

**Published:** 2022-07-18

**Authors:** Brandon Tan, Keren Zhou, Wei Liu, Emily Prince, Ying Qing, Yangchan Li, Li Han, Xi Qin, Rui Su, Sheela Pangeni Pokharel, Lu Yang, Zhicong Zhao, Chao Shen, Wei Li, Zhenhua Chen, Zheng Zhang, Xiaolan Deng, Andrew Small, Kitty Wang, Keith Leung, Chun-Wei Chen, Binghui Shen, Jianjun Chen

**Affiliations:** 1 Department of Systems Biology, Beckman Research Institute of City of Hope, Monrovia, CA 91016, USA; 2 Department of Cancer Genetics and Epigenetics, Beckman Research Institute of City of Hope, Duarte, CA91010, USA; 3 Gehr Family Center for Leukemia Research, City of Hope, Duarte, CA 91010, USA; 4 Department of Immunology, School of Basic Medicine, Hebei Medical University, 361 Zhongshan East Road, Shijiazhuang, Hebei 050017, China; 5 Department of Radiation Oncology, The First Affiliated Hospital of Sun Yat-sen University, Guangzhou, Guangdong 510080, China; 6 Department of Pharmacology, School of Pharmacy, China Medical University, Shenyang 110122, China; 7 Department of Liver Surgery, Renji Hospital, Shanghai Jiao Tong University School of Medicine, Shanghai 200127, China

**Keywords:** *N^6^*-methyladenosine, YTHDC1, TGF-β, SMAD3, metastasis

## Abstract

RNA *N^6^*-methyladenosine (m^6^A) modification and its regulators fine tune gene expression and contribute to tumorigenesis. This study aims to uncover the essential role and the underlying molecular mechanism(s) of the m^6^A reader YTHDC1 in promoting triple negative breast cancer (TNBC) metastasis.

Methods: *In vitro* and *in vivo* models were employed to determine the pathological function of *YTHDC1* in TNBC metastasis. To identify *bona fide* YTHDC1 target RNAs, we conducted RNA-seq, m^6^A-seq, and RIP-seq, followed by integrative data analysis and validation assays.

Results: By analyzing The Cancer Genome Atlas (TCGA) dataset, we found that elevated expression of *YTHDC1* is positively correlated with poor prognosis in breast cancer patients. Using a mammary fat pad mouse model of TNBC, *YTHDC1* significantly promoted lung metastasis of TNBC cells. Through multiple transcriptome-wide sequencing and integrative data analysis, we revealed dysregulation of metastasis-related pathways following YTHDC1 depletion and identified SMAD3 as a bona fide YTHDC1 target RNA. Depletion of YTHDC1 caused nuclear retention of *SMAD3* mRNA, leading to lower SMAD3 protein levels. Loss of YTHDC1 led to impaired TGF-β-induced gene expression, leading to inhibition of epithelial-mesenchymal transition (EMT) and suppressed TNBC cell migration and invasion. SMAD3 overexpression was able to restore the response to TGF-β in YTHDC1 depleted TNBC cells. Furthermore, we demonstrated that the oncogenic role of YTHDC1 is mediated through its recognition of m^6^A as m^6^A-binding defective mutants of YTHDC1 were unable to rescue the impaired cell migration and invasion of YTHDC1 knockout TNBC cells.

Conclusions: We show that YTHDC1 plays a critical oncogenic role in TNBC metastasis through promoting the nuclear export and expression of *SMAD3* to augment the TGF-β signaling cascade. Overall, our study demonstrates that YTHDC1 is vital for TNBC progression by enhancing TNBC cell survival and TGF-β-mediated EMT via SMAD3 to enable the formation of distant metastasis and highlights the therapeutic potential of targeting the YTHDC1/m^6^A/SMAD3 axis for TNBC treatment.

## Introduction

Breast cancer is the most frequently diagnosed cancer in the United States, making up 15% of all cancer diagnoses in 2021 1. Triple negative breast cancer (TNBC) patients with distant metastases only have a 5-year survival rate of 12%, due to its heterogenous and highly invasive nature coupled with a dearth of treatment options compared to other breast cancer subtypes 1, 2. Therefore, it is critical to better understand the molecular pathways and mechanisms that contribute to tumorigenesis and metastasis of TNBC and thereby identify novel therapeutic targets.

In normal cells, TGF-β signaling pathway plays a major role in driving organismal development, immune tolerance, and stem cell maintenance 3. Breast cancer cells exploit the TGF-β pathway to promote epithelial-mesenchymal transition (EMT) leading to elevated migratory and invasive characteristics to enhance metastasis to distant sites like the lungs and bones 4, 5. Serum levels of TGF-β are often elevated in breast cancer patients and is correlated to worse survival, underscoring the effects of the tumor microenvironment on metastasis 6. The canonical TGF-β pathway is activated by binding of extracellular TGF-β to the TGF-β receptor which then phosphorylates SMAD2 and SMAD3. Following this, SMAD2 and SMAD3 form a complex with a co-activator, SMAD4, and other transcription factors, culminating in the activation of TGF-β responsive genes which promote EMT 7. Thus, it is essential to better understand the mechanisms that augment the sensitivity of cells to TGF-β, which could lead to additional therapeutic avenues to treat metastatic TNBC.

*N^6^*-methyladenosine (m^6^A) is the most abundant internal chemical modification in eukaryotic messenger RNA (mRNA) which is able to fine-tune gene expression 8, 9. It is found within the RRACH motif and is associated with regulation of transcription, splicing, nuclear export, RNA stability, and translation 9-15. Importantly, m^6^A binding proteins (i.e., “readers”) bind to methylated transcripts and direct these transcripts to other complexes in the cell, thus governing the fate of methylated transcripts 8, 9, 16. The YT521 homology (YTH) domain family of proteins are the most well-studied m^6^A readers, and among them, *YTHDC1* has been linked to nuclear export, transcription, splicing, and RNA stability of methylated transcripts 12, 15, 17-19.

Dysregulated m^6^A methylation is a known contributor to tumorigenesis and metastasis of numerous cancers in part due to the upregulation or downregulation of various m^6^A “writers”, “erasers”, or “readers” 20-22. To date, *METTL3*, *ALKBH5*, *FTO*, and *YTHDF2* have been implicated in promoting breast cancer tumorigenesis by affecting cancer cell survival/proliferation and cancer stem cell pluripotency 23-26. *YTHDC1* has been shown to promote endometrial cancer, glioblastoma, and acute myeloid leukemia (AML) progression 27-31; however, its role in breast cancer remains unknown.

In the present study, we identify YTHDC1 as a novel facilitator of TNBC metastasis, with our *in vivo* animal model studies demonstrating a pivotal role for YTHDC1 in promoting lung metastasis. Mechanistically, YTHDC1 enhances nuclear export of *SMAD3* mRNA in an m^6^A-dependent manner to sensitize TNBC cells to the effects of TGF-β treatment.

## Results

### Elevated expression of *YTHDC1* predicts poorer survival in breast cancer patients

**Figure 1:**
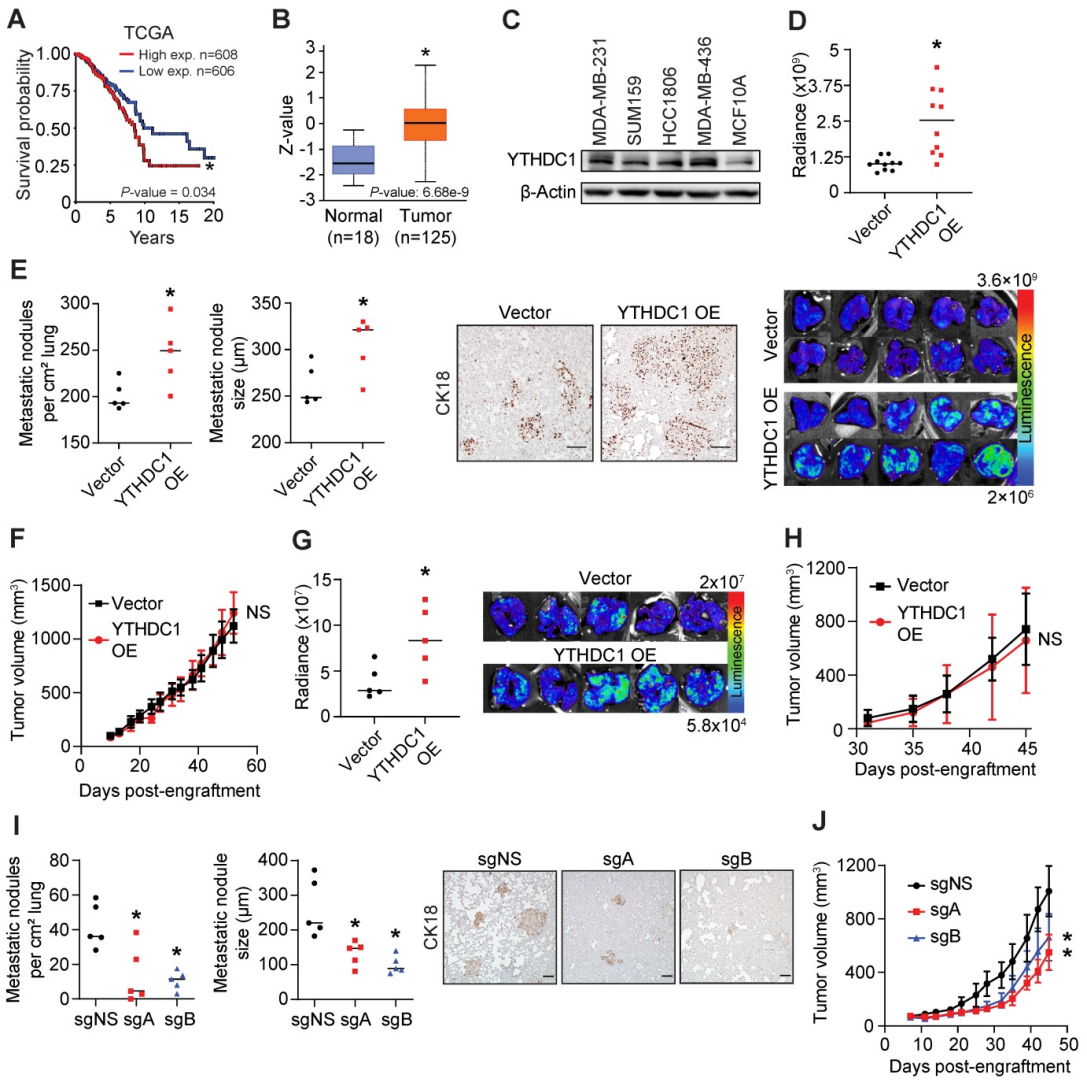
YTHDC1 promotes TNBC metastasis. **A)** Breast cancer patient survival in patients expressing high or low levels of YTHDC1 from TCGA data. Log-rank test.** B)** Box-whisker plot of YTHDC1 protein levels in tumor and normal patient samples from CPTAC data. Z-values represent standard deviations from the median across samples. *t*-test. **C)** Western Blot of YTHDC1 levels across different TNBC cells and non-tumorigenic MCF10A cells. **D-F)** Cells were engrafted into the 4^th^ mammary fat pad of 6-9 week-old NSG mice. 1×10^6^ MDA-MB-231 cells overexpressing YTHDC1 (OE) or vector were engrafted into each mouse. n = 10/group. **D)** Quantification of lung metastases by chemiluminescence (top) and associated images (bottom). *t*-test. **E)** Number and size of lung metastases (left) and corresponding images of lung samples (right) stained for human CK18. Each point represents the average number or size of 10 metastatic nodules in each mouse. Scale bar: 100 μm. n = 5/group. *t*-test. **F)** Volume of primary tumor measured twice weekly. Two-way ANOVA.** G-H)** 2×10^5^ SUM159 cells overexpressing YTHDC1 (OE) or vector were engrafted into each mouse. n = 5/group. **G)** Quantification of lung metastases by chemiluminescence and associated images. *t*-test. **H)** Volume of primary tumor measured twice weekly. Two-way ANOVA. **I-J)** 1×10^6^ MDA-MB-231 cas9 cells transduced with either control sgRNA (sgNS) or sgRNAs targeting YTHDC1 (sgA and sgB) were engrafted into each mouse. n = 5/group.** I)** Number and size of lung metastases and images of lung samples stained for human CK18. Each point represents the average number or size of 10 metastatic nodules in each mouse. n = 5/group. Scale bar: 100 μm. One-way ANOVA compared to sgNS. **J)** Volume of primary tumor measured twice weekly. Two-way ANOVA. The data are presented as the mean ± SD for tumor volume plots; NS = not significant, *P < 0.05.

We analyzed patient survival and *YTHDC1* gene expression data from The Cancer Genome Atlas (TCGA) cohorts and found that higher levels of *YTHDC1* in breast tumors resulted in a significantly poorer prognosis (Figure 1A). Mass spectrometry data from the Clinical Proteomic Tumor Analysis Consortium (CPTAC) showed that primary tumors express YTHDC1 protein at significantly higher levels compared to normal cells (Figure 1B). Notably, YTHDC1 protein expression is significantly higher in tumors having a proteomic profile representing upregulation of collagen VI (k7) and endoplasmic reticulum-related proteins (k10), which are consistent with cells undergoing metastasis/EMT, compared to either normal cells or tumors with a basal-like breast cancer proteomic profile (k4) 32-34 (Figure S1A). Higher YTHDC1 protein levels were observed in the TNBC cell lines MDA-MB-231, SUM159, HCC1806, and MDA-MB-436 compared to non-tumorigenic MCF10A mammary cells (Figure 1C). Taken together, both clinical and laboratory evidence imply an oncogenic role for YTHDC1 in TNBC.

### YTHDC1 promotes lung metastasis in a xenograft model

To determine the pathological role of YTHDC1 in TNBC, we utilized an NSG mouse model and engrafted MDA-MB-231 cells overexpressing *YTHDC1* in the 4^th^ mammary fat pad. Strikingly, mice engrafted with TNBC cells overexpressing *YTHDC1* developed significantly more lung metastases compared to control cells (Figure 1D). Immunohistochemistry staining of the lungs with anti-human CK18 antibody revealed greater numbers and larger sizes of metastatic nodules in the YTHDC1 overexpressing group (Figure 1E). Despite an increase in lung metastases, *YTHDC1* overexpressing MDA-MB-231 cells formed similar sized primary tumors to those in the control mice (Figure 1F and Figure S1B). We repeated this model using another TNBC cell line overexpressing *YTHDC1*, SUM159, and likewise found significantly higher levels of lung metastases without an increase in primary tumor size compared to control mice (Figure 1G, 1H, and Figure S1C). Conversely, *YTHDC1* KO in TNBC cells (MDA-MB-231) resulted in significantly decreased number and size of lung metastases in xenograft recipient mice (Figure 1I) and resulted in a moderate decrease in primary tumor size compared to control mice (Figure 1J and Figure S1D). Collectively, our *in vivo* animal model data support a tumor-promoting role for YTHDC1, which is consistent with clinical evidence. Notably, we found that YTHDC1 plays a greater role in promoting *in vivo* lung metastasis with a lesser effect on the growth of the primary tumor.

### YTHDC1 is a key regulator of pathways important for TNBC metastasis

**Figure 2:**
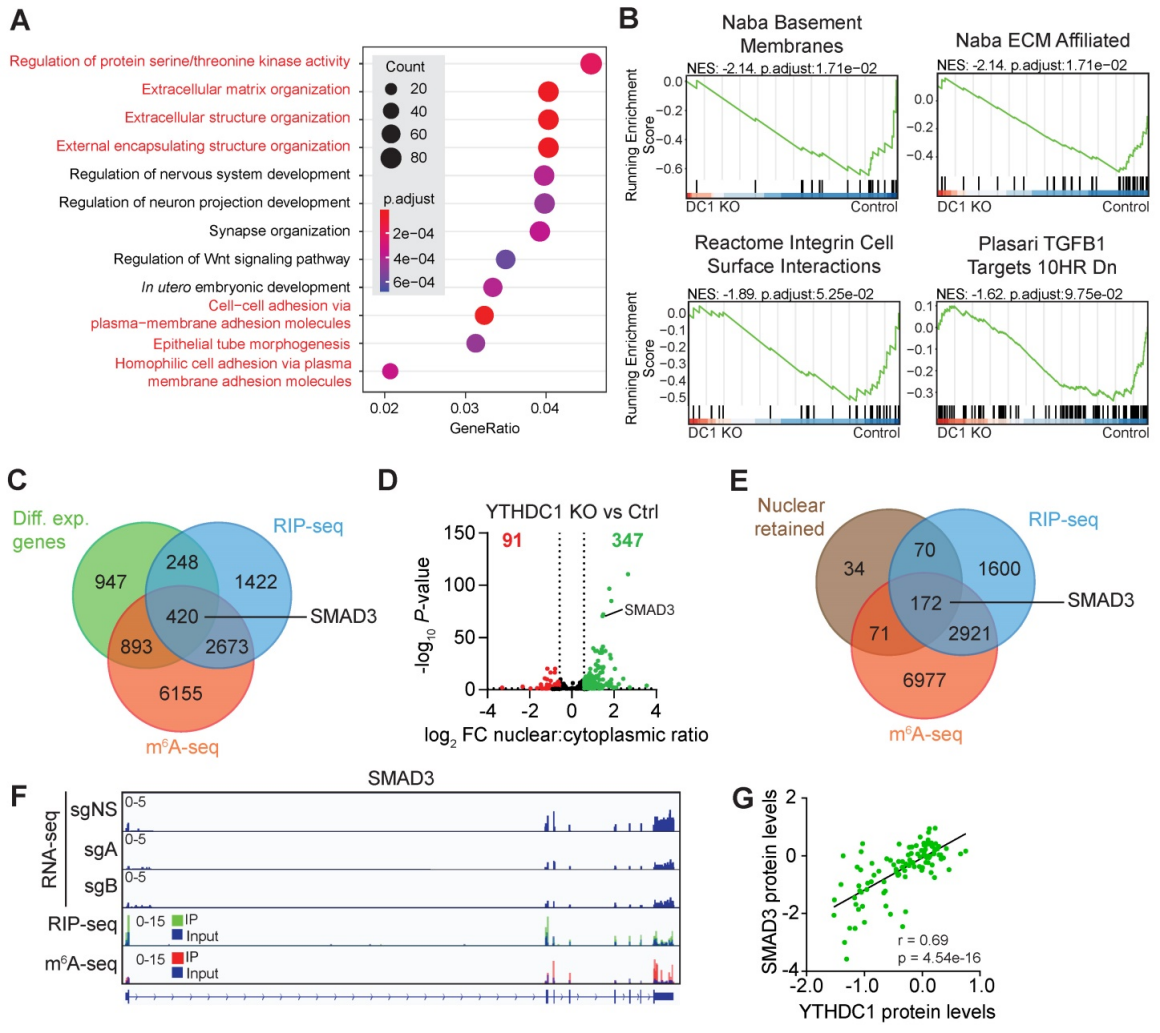
Identification of YTHDC1 target mRNAs in MDA-MB-231 cells by integrated analysis of sequencing data. **A)** Gene Ontology (GO) analysis of significantly differentially expressed genes in MDA-MB-231 YTHDC1 KO cells compared to control. Pathways related to metastasis are highlighted in red. **B)** Pathways related to metastasis enriched in Gene Set Enrichment Analysis (GSEA) of significantly differentially expressed genes in MDA-MB-231 YTHDC1 KO cells compared to control. **C)** Venn diagram of differentially expressed genes overlapped with RIP-seq and m^6^A-seq data. **D)** Volcano plot showing changes in mRNA nuclear export following YTHDC1 KO compared to control. **E)** Venn diagram of nuclear retained transcripts overlapped with RIP-seq and m^6^A-seq data. **F)** RNA-seq, RIP-seq and m^6^A-seq tracks for SMAD3. **G)** Correlation between SMAD3 protein and YTHDC1 protein expression levels from CPTAC breast cancer tumor samples. Pearson's correlation test.

To decipher the molecular mechanism by which YTHDC1 plays a critical role in TNBC metastasis, we conducted RNA-seq with poly(A) RNA from MDA-MB-231 *YTHDC1* KO (sgA and sgB) and control cells (Figure S2A). Differentially expressed genes enriched for pathways related to the extracellular matrix, cell-cell adhesion, and epithelial to mesenchymal transition (EMT) were found by Gene Ontology (GO) analysis (Figure 2A). Similarly, Gene Set Enrichment Analysis (GSEA) identified significantly enriched gene sets involved in metastasis such as basement membranes, the extracellular matrix, cell surface interactions, and transforming growth factor beta (TGF-β) (Figure 2B). These enriched pathways reflect YTHDC1's major role in promoting metastasis of TNBC cells as seen in our *in vivo* studies.

### *SMAD3* is a *bona fide* YTHDC1 target, whose nuclear export is regulated by YTHDC1 in TNBC cells

Next, we elected to identify high confidence targets of YTHDC1 by combining four sequencing strategies: m^6^A-seq, RNA-immunoprecipitation (RIP)-seq, whole cell RNA-seq, and nuclear vs cytoplasmic RNA-seq (Figure S2A-S2E). We utilized m^6^A-seq to identify m^6^A-methylated transcripts and RIP-seq to identify YTHDC1 bound transcripts in MDA-MB-231 cells. Since YTHDC1 has been reported to play a role in RNA stability and transcription 12, 27, 28, we focused on identifying YTHDC1 targets that were differentially expressed by overlapping RIP-seq, m^6^A-seq, and differentially expressed transcripts from RNA-seq. Among the 420 genes found was *SMAD3* (Figure 2C), a vital TGF-β-activated transcription factor that transactivates many genes involved in epithelial to mesenchymal transition (EMT), cell motility, and metastasis 3. Since YTHDC1 has also been shown to promote nuclear export of mRNA to the cytoplasm 19, we performed RNA-seq on nuclear and cytoplasmic fractions of MDA-MB-231 *YTHDC1* KO cells to identify differentially exported mRNA (Figure S2D and S2E). As expected, *YTHDC1* KO resulted in an increase in RNA nuclear retention, with 347 genes retained in the nucleus (Figure 2D). After cross-referencing this list with m^6^A-seq and RIP-seq data, we identified 172 potential YTHDC1 targets and strikingly, SMAD3 was also among the most significant transcripts retained in the nucleus (Figure 2D and 2E). Tracks of RNA-seq data visualize the downregulation of *SMAD3* following *YTHDC1* KO in MDA-MB-231 cells (Figure 2F). RIP-seq and m^6^A-seq tracks also show enrichment of YTHDC1 binding and the presence of m^6^A, respectively, on exons 1, 2, and 8 of *SMAD3* RNA (Figure 2F). In addition, CPTAC data showed a significantly positive correlation between the protein expression levels of YTHDC1 and SMAD3 in breast cancer patient samples, which supports regulation of SMAD3 by YTHDC1 (Figure 2G). Together, our data suggest *SMAD3* as a high confidence YTHDC1 target in TNBC.

**Figure 3:**
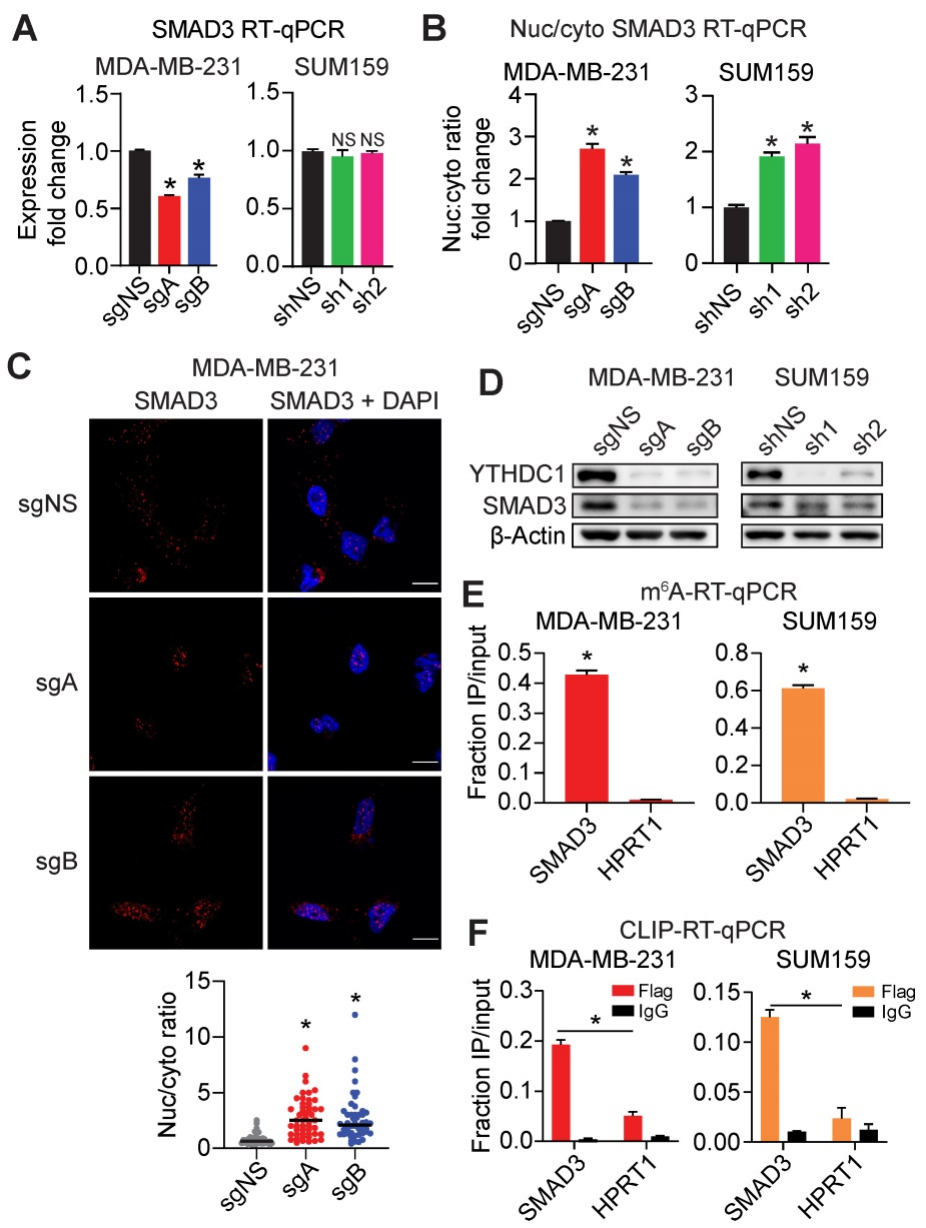
YTHDC1 promotes SMAD3 mRNA nuclear export in TNBC cells. **A)** SMAD3 transcript levels quantified by RT-qPCR in MDA-MB-231 YTHDC1 KO or SUM159 YTHDC1 KD cells. One-way ANOVA compared to control. **B)** Changes in the nuclear to cytoplasmic ratio of SMAD3 mRNA in MDA-MB-231 YTHDC1 KO or SUM159 YTHDC1 KD cells quantified by RT-qPCR. One-way ANOVA compared to control. **C)** Representative images and quantification of SMAD3 mRNA localization by FISH following YTHDC1 KO in MDA-MB-231 cells. Scale bar: 10 μm. Each point on the graph represents a single cell. One-way ANOVA compared to sgNS. n = 50/group. **D)** Western Blots of SMAD3 in MDA-MB-231 YTHDC1 KO or SUM159 YTHDC1 KD cells. **E)** m^6^A-RT-qPCR for SMAD3 in MDA-MB-231 and SUM159 cells. HPRT1 was used as a non-target control. One-way ANOVA compared to HPRT1. **F)** CLIP-RT-qPCR for SMAD3 in MDA-MB-231 and SUM159 cells. HPRT1 was used as a non-target control. One-way ANOVA compared to flag-immunoprecipitated HPRT1. RT-qPCR data are presented as the mean ± SD; n = 3/group, NS = not significant, *P < 0.05.

Following *YTHDC1* depletion in MDA-MB-231 and SUM159 cells, we observed only either a modest reduction (in MDA-MB-231 cells) or no significant change (in SUM159 cells) in *SMAD3* mRNA expression (Figure 3A). We then verified nuclear retention of *SMAD3* mRNA by nuclear/cytoplasmic fractionation followed by RT-qPCR and found a 2-3-fold elevation of SMAD3 mRNA in the nucleus following depletion of *YTHDC1* in MDA-MB-231 and SUM159 cells (Figure 3B and Figure S3A). In addition, we also observed increased nuclear retention of *SMAD3* mRNA by fluorescence *in situ* hybridization (FISH) upon *YTHDC1* depletion in MDA-MB-231 and SUM159 cells (Figure 3C and Figure S3B). At the protein level, the loss of YTHDC1 resulted in decreased SMAD3 protein in both MDA-MB-231 and SUM159 cells (Figure 3D). Additionally, forced expression of YTHDC1 caused a noticeable increased expression of SMAD3 protein in mouse tumors, and the opposite is true when YTHDC1 was knocked out (Figure S1B, S1C, and S1D). To validate m^6^A-seq data, we conducted m^6^A-RT-qPCR in both MDA-MB-231 and SUM159 cells and found significantly enriched levels of m^6^A on *SMAD3* mRNA compared to *HPRT1* which is an unmethylated control (Figure 3E). Crosslinking and immunoprecipitation (CLIP)-RT-qPCR in both MDA-MB-231 and SUM159 cells demonstrated significantly higher levels of *SMAD3* transcripts, compared to *HPRT1*, bound by flag-tagged-YTHDC1, thus validating RIP-seq data (Figure 3F and Figure S3C). In summary, direct binding of YTHDC1 to *SMAD3* mRNA results in its efficient nuclear export, thus augmenting SMAD3 protein expression in TNBC cells.

### YTHDC1 is essential for TGF-β-induced EMT of TNBC cells

**Figure 4:**
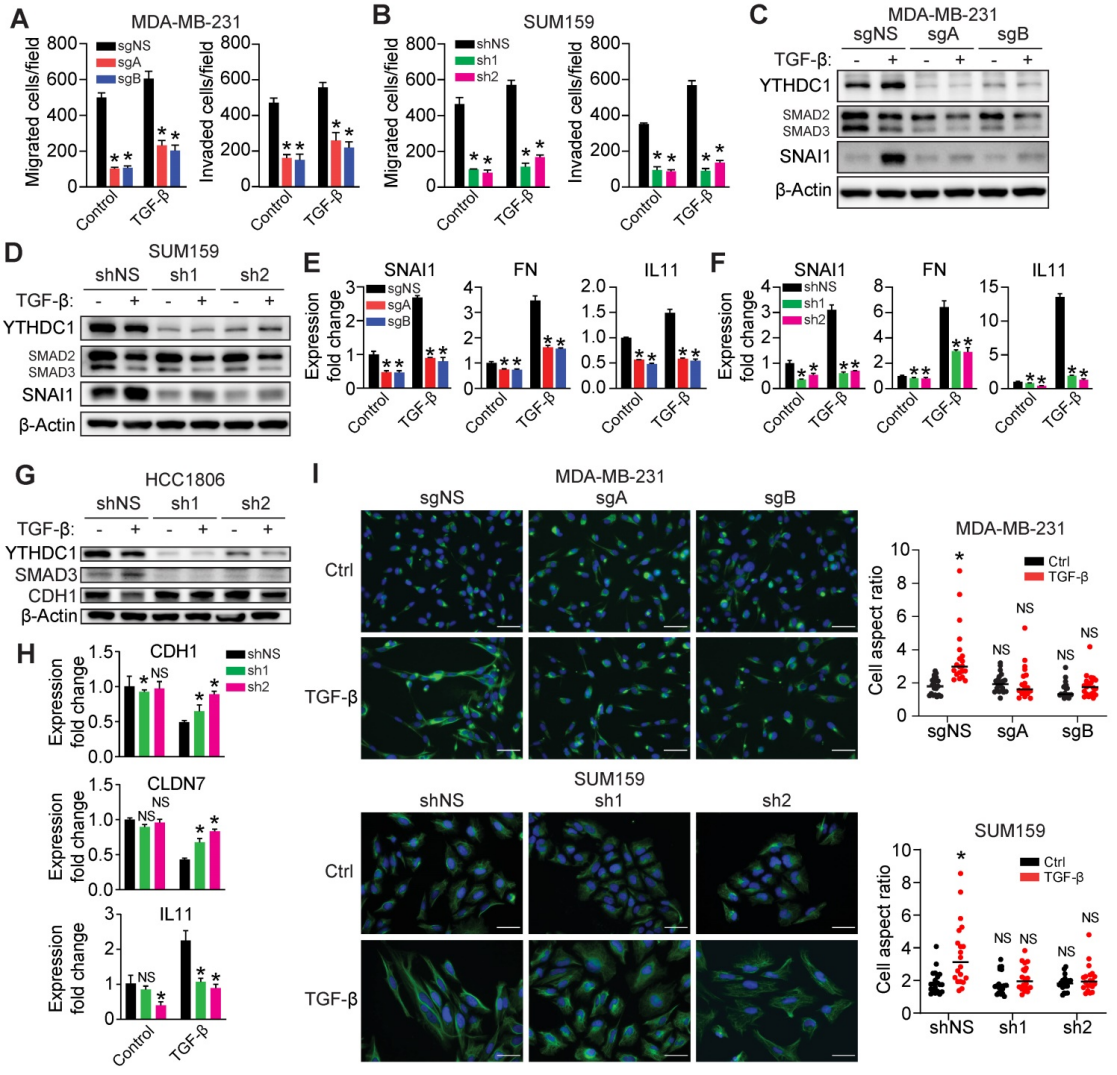
YTHDC1 is essential for TGF-β-mediated EMT.** A-B)** Transwell migration and invasion assays for **(A)** MDA-MB-231 YTHDC1 KO cells (5×10^4^ cells/well incubated for 5 hours for migration and 24 hours for invasion) or **(B)** SUM159 YTHDC1 KD cells (5×10^4^ cells/well incubated for 24 hours for migration and invasion) treated with 5 ng/ml TGF-β. One-way ANOVA compared to sgNS (MDA-MB-231) or shNS (SUM159) group. *P < 0.05. **C-D)** Western Blot for protein quantification from **(C)** MDA-MB-231 YTHDC1 KO cells or **(D)** SUM159 YTHDC1 KD cells treated with 5 ng/ml TGF-β for 24 hours. **E-F)** RT-qPCR of TGF-β responsive genes in **(E)** MDA-MB-231 YTHDC1 KO cells or **(F)** SUM159 YTHDC1 KD cells treated with 5 ng/ml TGF-β for 24 hours. One-way ANOVA compared to sgNS (MDA-MB-231) or shNS (SUM159) group. *P < 0.05. **G)** Western Blot for protein quantification from HCC1806 YTHDC1 KD cells treated with 5 ng/ml TGF-β for 72 hours. **H)** RT-qPCR of TGF-β responsive genes in HCC1806 YTHDC1 KD cells treated with 5 ng/ml TGF-β for 72 hours. One-way ANOVA compared to shNS group. NS = not significant, *P < 0.05. **I)** Images and quantification of the shapes of control, YTHDC1 MDA-MB-231 KO and SUM159 KD cells treated with TGF-β or vehicle control for 4 days and stained with vimentin. Scale bar: 50 μm. A larger aspect ratio represents a more elongated cell shape. One-way ANOVA compared to control shNS group. NS = not significant, *P < 0.05.

To test whether YTHDC1 is involved in fine-tuning TGF-β signaling, we treated MDA-MB-231 and SUM159 cells (with or without *YTHDC1* depletion) with 5 ng/ml TGF-β and measured cell migration and invasion, the expression of TGF-β-induced genes, and cell morphology. The loss of YTHDC1 abrogates TGF-β-induced Transwell migration and invasion of both cell lines (Figure 4A and 4B and Figure S3D and S3E). We observed a prominent lack of SNAI1 protein expression following 24 hours of TGF-β treatment in both *YTHDC1* KO MDA-MB-231 and *YTHDC1* KD SUM159 cells, indicating that this critical TGF-β-responsive gene is suppressed in YTHDC1 depleted cells (Figure 4C and 4D). Furthermore, we also saw decreased *SNAI1*, *FN*, and *IL11* RNA levels in YTHDC1 depleted MDA-MB-231 and SUM159 cells following TGF-β treatment (Figure 4E and 4F), indicating a perturbation in TGF-β signaling. In HCC1806 cells, loss of YTHDC1 increased levels of epithelial markers such as *CDH1* and *CLDN7* and suppressed the mesenchymal marker *IL11* following TGF-β treatment (Figure 4G and 4H). Additionally, the induction of an elongated, mesenchymal morphology associated with TGF-β treatment was suppressed in YTHDC1 depleted TNBC cells (Figure 4I). Taken together, our data highlight an essential role for YTHDC1 in promoting TGF-β-signaling and enhancing EMT and cell migration/invasion.

### Forced expression of SMAD3 restores migration and invasion of YTHDC1 depleted cells

**Figure 5:**
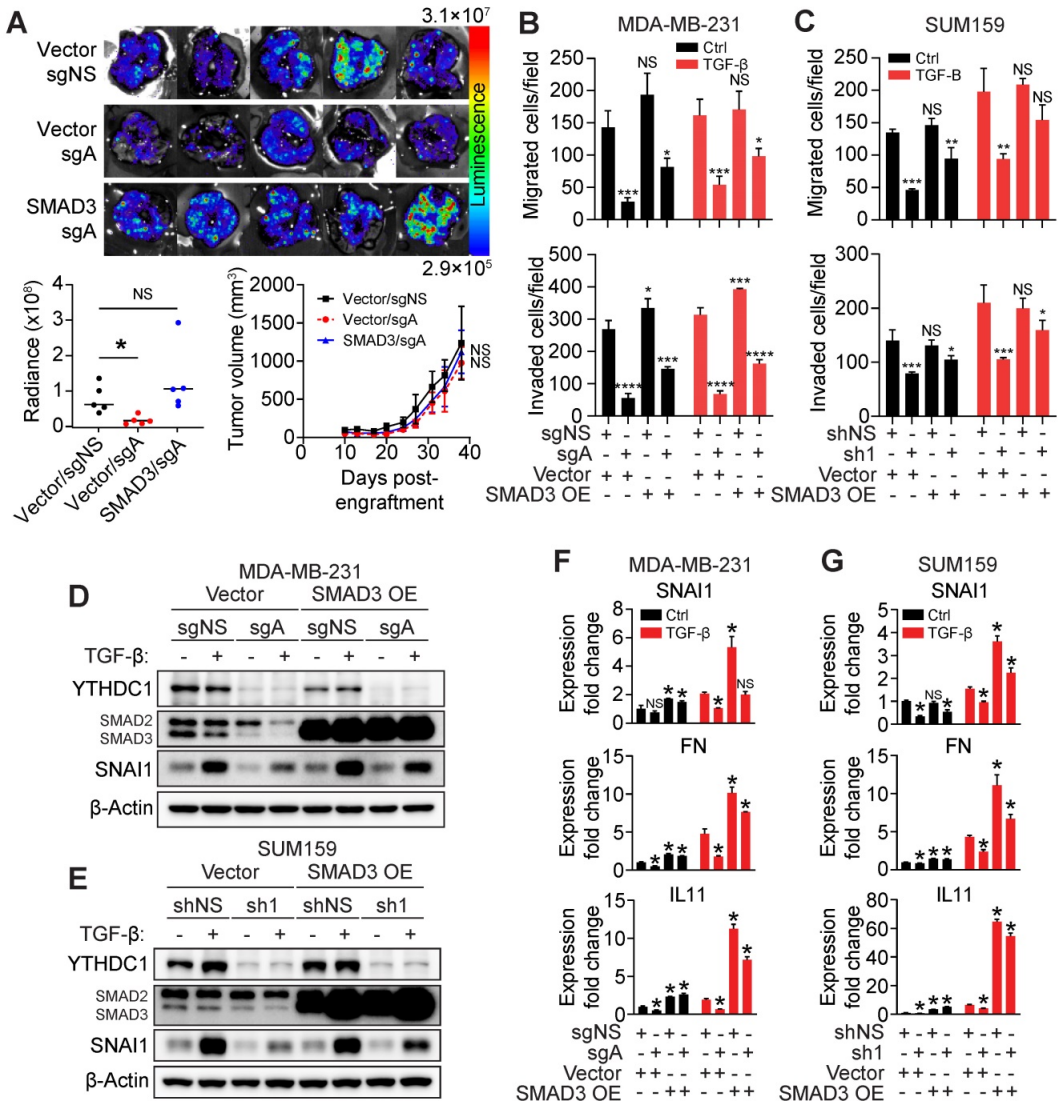
YTHDC1 promotes TGF-β-mediated metastasis by augmenting SMAD3 levels.** (A)** MDA-MB-231 cas9 cells were engrafted into the 4^th^ mammary fat pad of 6-9 week-old NSG mice. 2×10^6^ cells expressing either vector + sgNS, vector + sgA, or SMAD3 overexpression + sgA were engrafted into each mouse. n = 5/group. Associated lung images (top) and quantification of chemiluminescence signal (bottom left). One-way ANOVA compared to vector + sgNS group. NS = not significant, *P < 0.05. Tumor volumes are also shown (bottom right). Two-way ANOVA compared to vector + sgNS group. NS = not significant, *P < 0.05. **B-C)** Transwell migration and invasion assays for **(B)** MDA-MB-231 YTHDC1 KO cells (4×10^4^ cells/well incubated for 24 hours for migration and invasion) or **(C)** SUM159 YTHDC1 KD (4×10^4^ cells/well incubated for 24 hours for migration and invasion) cells overexpressing SMAD3 treated with 5 ng/ml TGF-β. One-way ANOVA compared to vector + sgNS/shNS group. NS = not significant, *P < 0.05, **P < 0.01, ***P < 0.001, ****P < 0.0001. **D-E)** Western Blot for protein quantification from **(D)** MDA-MB-231 YTHDC1 KO cells or **(E)** SUM159 YTHDC1 KD cells overexpressing SMAD3 treated with 5 ng/ml TGF-β for 24 hours. **F-G)** RT-qPCR of TGF-β responsive genes in **(F)** MDA-MB-231 YTHDC1 KO cells or **(G)** SUM159 YTHDC1 KD cells overexpressing SMAD3 treated with 5 ng/ml TGF-β for 24 hours. One-way ANOVA compared to vector + sgNS/shNS group. *P < 0.05. The data are presented as the mean ± SD; n = 3/group.

Given that SMAD3 is a critical downstream effector of TGF-β signaling, we hypothesized whether SMAD3 overexpression could rescue the effects of YTHDC1 depletion in TNBC cells. An *in vivo* rescue experiment was performed and we observed that overexpressing SMAD3 in MDA-MB-231 YTHDC1 KO cells restored metastasis in the lungs, with little effect on primary tumor size (Figure 5A and Figure S4A). *In vitro*, the impaired migration and invasion phenotypes seen in YTHDC1 depleted cells were partially rescued by SMAD3 overexpression in TNBC cells (Figure 5B and 5C and Figure S4B and S4C). SNAI1 protein level following TGF-β treatment was largely restored by forced expression of SMAD3 in YTHDC1 depleted TNBC cells both in cell culture and in mouse primary tumors (Figure 5D, 5E and Figure S4A). In addition, SMAD3 overexpression also sufficiently restored the expression of other TGF-β-responsive genes such as *FN* and *IL11* in TGF-β treated YTHDC1 depleted cells (Figure 5F and 5G). Thus, overexpressing SMAD3 in YTHDC1 depleted cells is able to restore TGF-β signaling, indicating that YTHDC1 promotes TNBC metastasis at least in part by regulating *SMAD3* expression.

### YTHDC1 promotes TNBC metastasis as an m^6^A reader

**Figure 6:**
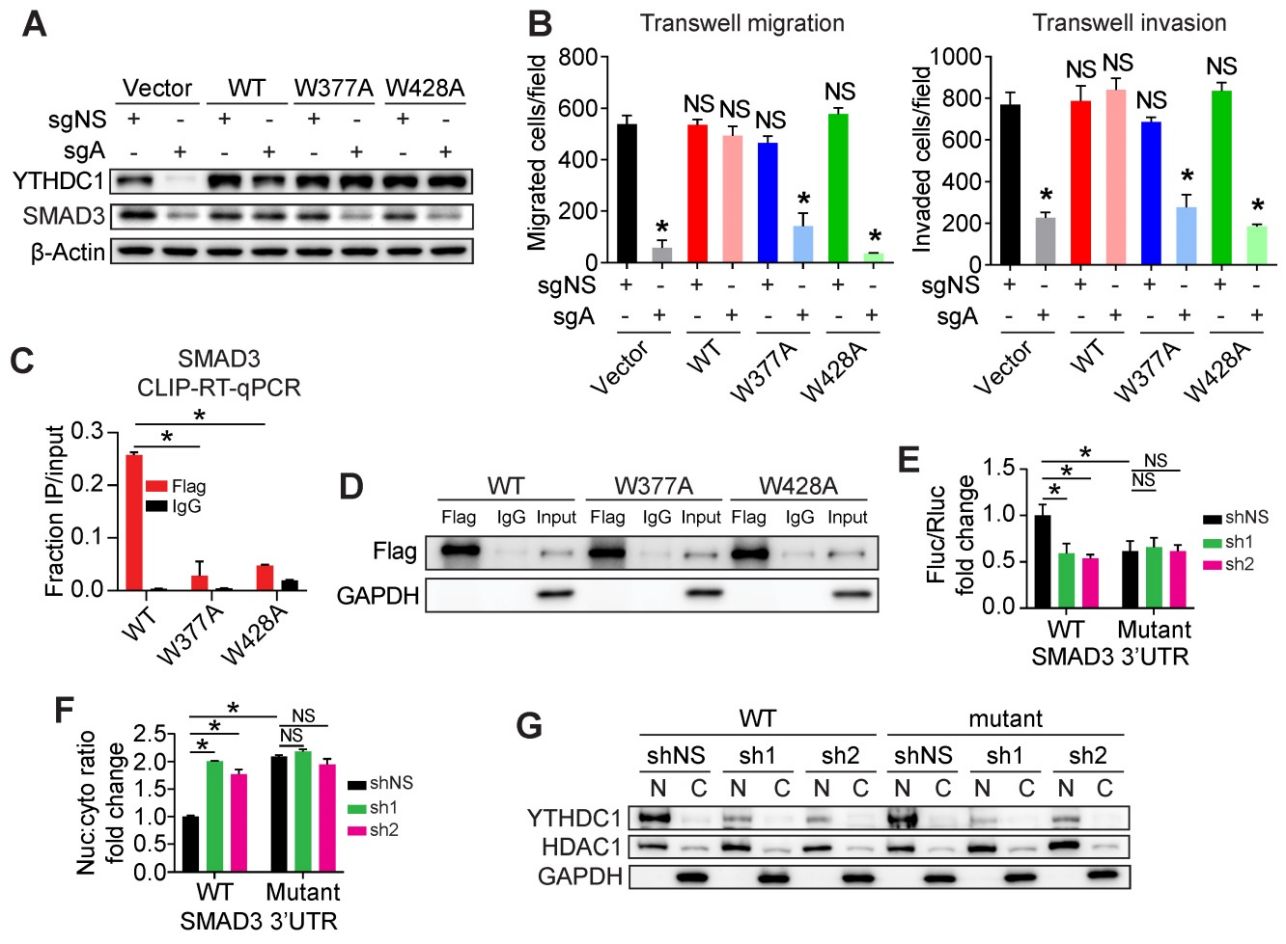
A functional m^6^A-binding domain is necessary for the tumorigenic functions of YTHDC1. **A)** Western Blot analysis of YTHDC1 and SMAD3 in MDA-MB-231 control or YTHDC1 KO cells overexpressing either empty vector, wild type (WT), or m^6^A-binding defective mutants (W377A and W428A) of YTHDC1. **B)** Transwell migration and invasion assays of MDA-MB-231 control (sgNS) or YTHDC1 KO cells overexpressing either empty vector, wild type (WT), or mutant (W377A or W428A) YTHDC1 (5×10^4^ cells/well incubated for 24 hours for migration and invasion). One-way ANOVA compared to Vector/sgNS group. **C)** CLIP-RT-qPCR of SMAD3 bound to wild type (WT) or mutant (W377A or W428A) YTHDC1 in MDA-MB-231 cells. One-way ANOVA compared to WT group. **D)** Western Blot for immunoprecipitation of wild type or mutant YTHDC1 using anti-flag or control IgG antibodies in CLIP-RT-qPCR assay. **(E)** Dual luciferase assay in HEK293T cells transfected with WT or mutant *SMAD3* 3'UTR fused to a firefly luciferase (Fluc) gene. Renilla luciferase (Rluc) used as a loading control. One-way ANOVA compared to shNS+WT *SMAD3* 3'UTR. **(F)** Changes in the nuclear to cytoplasmic ratio of firefly luciferase mRNA in HEK293T cells quantified by RT-qPCR. One-way ANOVA compared to shNS+WT *SMAD3* 3'UTR. The data are presented as the mean ± SD; n = 3/group. NS = not significant, *P < 0.05.** (G)** Western Blot assay showing nuclear and cytoplasmic distribution of YTHDC1 protein in HEK293T cells and YTHDC1 KD efficiency. HDAC1 and GAPDH were used as nuclear and cytoplasmic marker, respectively.

To determine whether YTHDC1's ability to bind to m^6^A is required for its oncogenic functions, we constructed two YTHDC1 point mutants, W377A and W428A, that have been shown to inhibit its binding to m^6^A 35. We then overexpressed wild type (WT) YTHDC1 or the W377A and W428A mutants in MDA-MB-231 YTHDC1 KO cells to determine if these mutants were able to rescue YTHDC1 target gene expression and tumorigenic phenotypes (Figure 6A). As expected, overexpression of WT but not the W377A and W428A YTHDC1 mutants was able to restore SMAD3 protein levels in MDA-MB-231 *YTHDC1* KO cells (Figure 6A). Furthermore, unlike WT YTHDC1, the W377A and W428A mutants could not rescue the impaired migration and invasion of MDA-MB-231 *YTHDC1* KO cells, demonstrating that the metastatic phenotype is dependent on m^6^A-binding by YTHDC1 (Figure 6B and Figure S4D and S4E). To demonstrate that the phenotypes observed were indeed due to direct binding of YTHDC1 to target transcripts, we performed CLIP-RT-qPCR in MDA-MB-231 cells overexpressing either Flag-tagged WT, W377A or W428A mutants of YTHDC1. We observed significantly decreased binding of *SMAD3* RNA to both YTHDC1 mutants compared to WT YTHDC1 (Figure 6C and 6D).

Our m^6^A-seq data showed that the first 800 bp of the SMAD3 3'UTR contains the majority of m^6^A modifications on SMAD3 mRNA (Figure 2F). All the 18 RRACH motif sites in this region were mutated to RRTCH and either the mutant or WT SMAD3 3'UTR was fused to a firefly luciferase gene. Using a dual luciferase assay, we observed that firefly luciferase activity of the mutant SMAD3 3'UTR was significantly decreased compared to that of the WT group in cells without *YTHDC1* KD, and the mutations in *SMAD3* 3'UTR abolished *YTHDC1* KD-mediated further down-regulation of the firefly luciferase activity (Figure 6E), indicating the importance of these m^6^A sites in *SMAD3* 3'UTR in YTHDC1-meidated promotion of SMAD3 expression. Furthermore, mutant *SMAD3* 3'UTR caused nuclear retention of firefly luciferase mRNA compared to WT *SMAD3* 3'UTR (Figure 6F and 6G). This demonstrates that m^6^A on the *SMAD3* 3'UTR is recognized by YTHDC1 and results in efficient nuclear export of *SMAD3* mRNA. Taken together, these studies provide compelling evidence that YTHDC1's role in promoting TNBC cell migration and invasion is dependent on its ability to read m^6^A on SMAD3 mRNA.

## Discussion

Research into the tumorigenic role of m^6^A in breast cancer has linked m^6^A-regulators such as *METTL3*, *ALKBH5*, *FTO*, and *YTHDF2* to many facets of breast cancer tumorigenesis such as cell proliferation, survival, and cancer stem cell pluripotency 23-26, 36. In this study, we unravel *YTHDC1's* role as a significant contributor to TNBC metastasis which reflects the poor prognosis of patients expressing high levels of *YTHDC1*. *In vivo,* YTHDC1 promotes lung metastasis from the mammary fat pad despite having a limited effect on primary tumor size. Similar to our observation, previous studies reported that genes involved in migration and invasion such as *TGFBR1* and *EPHA1* also had a greater effect on tumor dissemination rather than on primary tumor size 5, 37. Further supporting this observation, our RNA-seq data identified significantly dysregulated pathways affecting cell migration, invasion, and metastasis in *YTHDC1* KO TNBC cells. Therefore, we uncover a previously unappreciated role of YTHDC1 in promoting TNBC metastasis.

We presumed that YTHDC1 has a wide repertoire of target transcripts as YTHDC1 has been implicated in diverse mechanisms, among them promoting RNA nuclear export by interacting with SRSF3 and enhancing RNA stability by sequestering RNA away from the polyA tail exosome targeting complex (PAXT) and exosome-associated RNA degradation 19, 27. Therefore, we searched for differentially expressed and differentially exported RNAs upon *YTHDC1* KO in TNBC cells by RNA-seq, RIP-seq, and m^6^A-seq to identify high confidence YTHDC1 targets such as *SMAD3*. Consistent with our discovery, previous studies have shown that m^6^A writer (METTL14) and eraser (ALKBH5) also modulate *SMAD3* expression 38, supporting our conclusion that *SMAD3* is a *bona fide* target of the m^6^A machinery. Specifically, we showed that YTHDC1 promotes nuclear export of methylated *SMAD3* mRNA and thereby affects its protein production. Strikingly, unlike MDA-MB-231 cells where YTHDC1 depletion caused a decrease in both nuclear export and levels of *SMAD3* mRNA, loss of YTHDC1 in SUM159 cells caused a decrease in nuclear export of *SMAD3* mRNA but did not affect the *SMAD3* mRNA stability. Therefore, the main mechanism utilized by YTHDC1 to regulate *SMAD3* expression in TNBC cells is nuclear export. Since previous studies demonstrated that elevated nuclear RNA export is a hallmark of breast cancer cells, in part due to upregulation of SRSF3 in HER2^+^ and triple negative breast cancers 39, our study suggests that YTHDC1 may act in concert with this mechanism to promote the expression of pro-metastatic genes such as *SMAD3*.

While YTHDC1 plays a role in elevating SMAD3 levels to initiate response of TNBC cells to TGF-β treatment, YTHDC1 may also promote the RNA stability, transcription, or nuclear export of critical downstream TGF-β-responsive genes thus strengthening the TGF-β response. In human pluripotent stem cells (hPSCs), SMAD2/3 was shown to interact with the m^6^A methyltransferase complex resulting in hypermethylation of downstream *SMAD2/3* target mRNAs such as *NANOG* and *SMAD7* 40. Another study showed that the loss of METTL14 caused reduced expression of downstream TGF-β genes in breast cancer cells 38. Therefore, this could explain why overexpression of SMAD3 alone does not fully rescue cell migration and invasion of YTHDC1 KO cells *in vitro* as YTHDC1 could also promote the expression of a set of TGF-β-responsive genes.

In conclusion, our findings contribute to the complexity of TGF-β signaling by linking *SMAD3* mRNA nuclear export to the m^6^A reader YTHDC1, thus promoting TGF-β-responsiveness, EMT, and metastasis of TNBC cells. Given the fundamental role TGF-β plays in metastasis, our findings could potentially be extended to additional invasive cancers such as other breast cancer subtypes, gliomas, and melanomas as intact SMAD signaling machinery is present in most of these cancer types 3. Furthermore, our work provides a rationale for the development of small molecule inhibitors targeting YTHDC1 as a therapeutic strategy to treat breast cancer and other cancers reliant on YTHDC1 such as AML, endometrial cancer, and glioblastoma.

## Methods

### Database analysis

YTHDC1 RNA-seq expression was correlated to patient survival using data from The Cancer Genome Atlas (TCGA) plotted in XenaBrowser 41. Clinical Proteomic Tumor Analysis Consortium (CPTAC) mass spectrometry data from normal and breast tumor samples and the different proteomic profiles in breast tumors were obtained from UALCAN 42.

### Cell culture

MDA-MB-231 and SUM159 cells were kind gifts from Dr. Jun-Lin Guan and Dr. Susan Waltz, respectively. Cells were authenticated using short tandem repeat analysis (Laragen, Inc.). MDA-MB-231 and HEK293T cells were cultured in DMEM supplemented with 10% FBS and 1% penicillin/streptomycin. SUM159 and HCC1806 cells were cultured in RPMI supplemented with 10% FBS and 1% penicillin/streptomycin. MCF10A cells (ATCC) were cultured in MEGM Mammary Epithelial Cell Growth Medium (CC-3150, Lonza). HEK293T cells used for lentivirus production were cultured in DMEM supplemented with 10% FBS and 1% penicillin/streptomycin. All cells were kept in a 37°C, 5% CO_2_ humidified incubator and were routinely tested for mycoplasma contamination using Mycoplasma PCR Detection Kit (Applied Biological Materials) and only mycoplasma free cells were used in experiments. An MDA-MB-231 CRISPR-cas9 single cell clone was isolated by plating lentiCas9-Blast (52962, Addgene) transduced cells into single cells in 96-well plates and selecting with 5 μg/ml of blasticidin.

### Plasmids, sgRNAs, and shRNAs

Overexpression plasmids were constructed using the pCDH puromycin resistant or PLX304 blasticidin resistant vectors. Overexpression sequences of YTHDC1, SPRY1, TUG1, and SMAD3 were cloned by PCR from cDNA of MDA-MB-231 cells. The W377A and W428A mutants of YTHDC1 were constructed using the Q5® Site-Directed Mutagenesis Kit (New England Biolabs). For rescue experiments using WT or mutant YTHDC1, the coding sequence was altered to carry synonymous mutations within the sgA site to prevent targeting by sgA. A full list of overexpression vectors is available in Table S1. sgRNA sequences were cloned into the lentiGuide-Puro vector. The sgRNA sequences used were YTHDC1 sgA: 5'-AGATGAGCCGACTTAGTGAA-3' and YTHDC1 sgB: 5'-ACGGAGGATCTCCTATACAC-3'. shRNA targeting *YTHDC1* were obtained from Sigma-Aldrich (sh1: TRCN0000243989, sh2: TRCN0000243987). Plasmids used for dual luciferase assay were obtained from Promega. The first 800bp of WT *SMAD3* 3'UTR was cloned from DNA isolated from MDA-MB-231 cells and fused to a firefly luciferase gene (E1751, Promega). All the 18 RRACH motif sites within the first 800 bp of *SMAD3* 3'UTR were mutated to RRTCH by site-directed PCR mutagenesis and fused to a firefly luciferase gene to generate mutant *SMAD3* 3'UTR. As a control, pRL-TK (E2241, Promega) expressing renilla luciferase was used.

### Lentiviral production

HEK293T cells were transfected with psPAX2, PMD2.G and the target vector using Transporter 5 transfection reagent (Polysciences) with a 3:1 (volume:mass) ratio of transfection reagent to plasmid DNA. The media was replaced 8 hours post-transfection and the supernatant was collected at 48- and 72-hours post-transfection. The supernatants from both time points were pooled and filtered through a 0.45μm syringe filter before being aliquoted and frozen at -80°C until use. Virus was titrated in MDA-MB-231 cells to determine the concentration of viral particles used to calculate multiplicity of infection (MOI).

### Lentiviral transduction

MDA-MB-231 or SUM159 cells were transduced with lentivirus using a MOI of 4. Lentivirus was added to cells in media containing 8 μg/ml polybrene (Sigma-Aldrich) and centrifuged at 1500rpm for 1 hour at room temperature. The media was replaced 24 hours later and supplemented with either 2 μg/ml puromycin or 5 μg/ml blasticidin, or both, depending on the required selection antibiotics. For YTHDC1 loss of function experiments, MDA-MB-231 cas9 or SUM159 cells were transduced with lentivirus carrying either sgRNA or shRNA, respectively. MDA-MB-231 cas9 YTHDC1 knockout (KO) cells were selected using puromycin for 4 days whereas SUM159 YTHDC1 knockdown (KD) cells were selected with puromycin for 2 days before experiments were performed. For rescue experiments, cells were transduced with the SMAD3 overexpression vector and selected for 7 days with blasticidin. The cells were then replated into 6-well plates and transduced with sgRNA or shRNA and selected with puromycin and blasticidin for either 4 or 2 days, respectively, before being used in further experiments.

### Western Blotting

Following trypsinization, cells were washed once with PBS and pelleted by centrifugation at 800 rpm for 5 minutes. The cell pellet was lysed with RIPA buffer (Sigma-Aldrich) supplemented with Halt^TM^ protease and phosphatase inhibitors (Thermo Fisher Scientific) and incubated on ice for 20 minutes. The lysate was cleared by centrifugation and protein concentration was determined using the Bio-Rad Protein Assay Kit (Bio-Rad). For each sample, 20 μg of protein was separated using a 10% SDS-PAGE gel and transferred onto a PVDF membrane (Thermo Fisher Scientific). Membranes were blocked with 5% skim milk in PBST and incubated with primary antibody. Goat anti-mouse-HRP (ab6789, Abcam) or anti-rabbit-HRP (ab6721, Abcam) were used as secondary antibodies. Substrate was added to the blots and imaging performed on a ChemiDoc MP (Bio-Rad) imager. Primary antibodies used for Western blotting were: YTHDC1 (A305-096A, Bethyl Laboratories), β-Actin (3700S, Cell Signaling Technologies), SMAD2/3 (3102S, Cell Signaling Technologies), SNAI1 (3895S, Cell Signaling Technologies), CDH1 (PA5-80457, Thermo Fisher Scientific), HDAC1 (sc-7872, Santa Cruz Biotechnology), Flag (F3165, Sigma-Aldrich), and GAPDH (10494-1-AP, Proteintech).

### Transwell migration and invasion assays

Transwell chambers with 8 μm pore size in 24-well plates (3422, Corning) were used for both migration and invasion assays. For invasion, 30 μg matrigel (356234, Corning) diluted in serum free media was used to coat the top of each Transwell and was left to solidify at 37°C for 2 hours before the cells were layered onto the gel. MDA-MB-231 or SUM159 cells were seeded in 200 μl serum free media above the Transwell membrane at the indicated cell concentration. 750 μL media with 10% FBS as a chemoattractant was added to the bottom of the well and cells were incubated for the indicated duration. For TGF-β experiments, the top and bottom layers of Transwells were supplemented with 5 ng/ml TGF-β (100-21, Peprotech).

### Animal experiments

NOD-SCID IL2Rγ-null (NSG, Jackson 005557) mice were originally purchased from the Jackson Laboratory and bred in accordance to the Institutional Animal Care and Use Committee (IACUC) protocols approved by City of Hope. All mice were grouped housed on a 12 hour:12 hour, light:dark cycle with food and water *ad libitum*. For each experiment, similar aged (6-9 weeks) mice were randomly assigned to experimental groups.

### Mammary fat pad engraftment of cells

MDA-MB-231 and SUM159 were transduced with pLenti-CMV-Puro-LUC (17477, Addgene) to generate luciferase expressing cells and selected with 2 μg/ml of puromycin for 4 days. Cells were then either transduced with *YTHDC1* or *SMAD3* overexpressing lentivirus or *YTHDC1* sgRNA/shRNA lentivirus and their respective controls. Cells resuspended in 100μL PBS were injected into the 4^th^ mammary fat pad of NSG mice. Tumors were measured with calipers twice weekly and tumor volume was calculated using the formula: (width^2^ × length) / 2. All mice were euthanized at the same time when the primary tumor volume exceeded 1500 mm^3^ or when tumors showed signs of ulceration.

### Bioluminescence imaging of lung metastases

Both the left and right lungs were removed from each mouse and laid out on a petri dish. 50 μL of luciferin substrate (LUCK-2G, Goldbio) was pipetted onto each lung prior to imaging on a Lago X Imaging System (Spectral Instruments). Radiance (watts per square meter per steradian) was measured from the images using Aura Imaging Software (Spectral Instruments) by utilizing an identical selection size for each lung in all images.

### CK18 immunohistochemistry staining of lung samples

Dissected lung tissue was incubated in 4% formaldehyde for 48 hours at room temperature, dehydrated using ethanol, and embedded in a paraffin block. Sections of tissue were made and mounted onto glass slides. The sections were deparaffinized with two changes of xylene and then rehydrated by incubation in decreasing concentrations of ethanol (100%, 95%, 70%, and 50%). Antigen retrieval was performed by boiling the slides in Tris-EDTA buffer pH 9.0 in a pressure cooker for 10 minutes. Endogenous peroxidase activity was blocked with 3% H_2_O_2_ and the slides were rinsed with PBS followed by blocking with 10% FBS in PBS for 1 hour at room temperature. The slides were stained with anti-human CK18 antibody (ab133263, Abcam, 1:100 dilution) for one hour at room temperature. Following two PBS washes, slides were incubated with goat anti-rabbit HRP secondary antibody (ab6721, Abcam, 1:200 dilution) at room temperature for 30 minutes. Slides were washed three times with PBS and DAB substrate was added to visualize human CK18. Slides were imaged using an Olympus BX61 imaging system.

### Nuclear/cytoplasmic separation of cells

One million cells were incubated in 100 μl hypotonic buffer (10mM Tris pH 7.4, 1.5mM MgCl_2_, 10mM KCl, 1% NP-40) on ice for 10 minutes to separate intact nuclei from cytoplasmic contents. The nuclei were pelleted by centrifugation at 5,000 × g at 4°C for 10 minutes and the supernatant containing the cytoplasmic fraction was collected. The nuclear pellet was washed once with 100 μl hypotonic buffer, spun down, and resuspended in 100 μl hypotonic buffer. RNA was isolated from half of the cytoplasmic and nuclear fractions using Trizol, and the other half of the fraction was incubated with 16.7 μl 4x Laemli buffer for protein extraction.

### RNA extraction from cells for RT-qPCR, RNA-seq, and m^6^A-seq

Cells were trypsinized, transferred to 1.5 ml tubes, and pelleted by centrifugation. 1 ml of Trizol (Qiagen) was added to each sample and the cells were lysed by vortexing. After incubation at room temperature for 10 minutes, 200 μl of chloroform was added and the solution was thoroughly mixed by shaking. Then, the tubes were centrifuged at 12,000 × g for 15 minutes at 4°C and 500 μl of the aqueous phase was transferred to another 1.5 ml tube. An equal amount of isopropanol was added followed by thorough mixing. The tubes were centrifuged at 12,000 × g for 15 minutes at 4°C and the supernatant was discarded. The pellet was washed with 1 ml of 75% ethanol and then pelleted by centrifugation at 12,000 × g for 5 minutes at 4°C. The supernatant was carefully discarded, and the pellet was left to air dry. Nuclease-free water (Thermo Fisher Scientific) was then used to resuspend the RNA pellet and its concentration was determined using a NanoDrop One (Thermo Fisher Scientific).

### mRNA isolation

PolyATtract® mRNA Isolation Systems kit (Promega) was used to isolate mRNA from purified total RNA following manufacturer's instructions.

### m^6^A-IP for m^6^A-RT-qPCR and m^6^A-seq

Purified mRNA was precipitated with 1:10 volume of 3M sodium acetate (pH 5.2) and 2.5 volumes of 100% ethanol at -80°C overnight. The next day, the mRNA was centrifuged at 15,000 × g for 15 minutes at 4°C and the supernatant was discarded. The pellet was washed with 1mL 75% ethanol and centrifuged at 15,000 × g for 5 minutes at 4°C. The supernatant was discarded and the pellet was left to air dry before being resuspended in nuclease-free water. The concentration was determined using a NanoDrop One (Thermo Fisher Scientific) and adjusted to 1 ug/ul with nuclease-free water. The mRNA was divided into 18uL aliquots in thin-walled PCR tubes. 2 μL of 10× fragmentation buffer (100mM ZnCl_2_ in 100mM Tris-HCl pH 7.0) was added and tubes were incubated at 94°C for exactly 4 minutes. Immediately, 2 μl of 500mM EDTA was added to each tube and put on ice. The fragmented mRNA was then purified by precipitation as outlined above before immunoprecipitation. At least 100 ng of fragmented RNA was retained as the input fraction. 50 μl of Protein A/G magnetic bead slurry (Thermo Fisher Scientific) were added to a 1.5 ml tube and washed twice with 1 ml of cold RNase-free PBS. Then, 5 μg of anti-m^6^A antibody (202003, Synaptics Systems) was incubated with the beads for 3 hours at 4°C. The beads were washed 3× with cold PBS and then once with IP buffer (10mM Tris-HCl pH 7.4, 750 mM NaCl and 0.5% (vol/vol) Igepal CA-630). A total of 10 μg of fragmented mRNA was incubated with the beads overnight at 4°C. The next day, the beads were washed 5× with IP buffer. To elute bound RNA, 100 μl of elution buffer (6.67mM m^6^A salt (Sigma-Aldrich) in IP buffer) was incubated with the beads for 1 hour at 4°C and the supernatant containing immunoprecipitated RNA was collected. The elution was repeated with another 100 μl of elution buffer. The eluted RNA was purified using the RNeasy Mini Kit (Qiagen) and eluted to a final volume of 15 μl. For m^6^A-RT-qPCR, 3 μl of immunoprecipitated eluate was used and 1% (1 ng) of the input amount used in each RT reaction. For m^6^A-seq, an Agilent Bioanalyzer 2100 was used to determine the size and quality of both IP and input samples prior to RNA library construction using KAPA Stranded mRNA-Seq Kit (Illumina Platforms) (KR0960m Kapa Biosystems) and sequenced on an Illumina Hiseq2500.

### Crosslinking and Immunoprecipitation (CLIP)-RT-qPCR and RIP-seq

MDA-MB-231 cells overexpressing flag-tagged-YTHDC1 or its mutants were grown to 80% confluence in 15cm tissue culture dishes. Cells were washed with cold PBS and UV-crosslinked 3× at 120,000 μJ/cm^2^ using a 254nm lamp. Nuclear isolation buffer (320mM sucrose 10mM Tris-HCl pH 7.5, 5mM MgCl_2_, 1% Triton X-100 vol/vol) was added onto the dish and the cells were detached with a cell scraper. The cells were incubated on ice for 20 minutes on ice followed by pelleting of nuclei by centrifugation at 1500 × g for 15 minutes at 4°C. The pellet was resuspended in RIP buffer (150mM NaCl, 25mM Tris-HCl pH 7.4, 5mM EDTA, 0.5mM DTT, and 0.5% Tergitol NP40) containing Halt^TM^ Protease (Thermo Fisher Scientific) and RiboLock (Thermo Fisher Scientific) RNase inhibitors and sonicated using a Qsonica Q800R3 sonicator to isolate nuclear contents. The lysate was centrifuged at 15,000 × g for 10 minutes at 4°C and the supernatant was pre-cleared by incubation with protein A/G beads for 1 hour at 4°C. The amount of protein in each sample was quantified using the Bio-Rad Protein Assay Kit (Bio-Rad). For each immunoprecipitation reaction, 5 μg of anti-flag antibody (F3165, Sigma-Aldrich) or normal mouse IgG (12-371, Millipore) was conjugated to 50 μl Protein A/G magnetic beads (Thermo Fisher Scientific) for 4 hours at 4°C. The beads were washed twice with RIP buffer and incubated with supernatant containing 500 μg of protein overnight at 4°C. Meanwhile, supernatant containing 50 μg of protein (10% input) was saved for each RNA or protein input sample. The Trizol extraction method described above was used to purify input RNA, which was resuspended in a final volume of 50 μl using RNase free water. Laemli buffer was added to input protein and denatured for 5 minutes at 95°C. Both RNA and protein input samples were kept at -80°C until needed. The next day, the immunoprecipitation reactions were washed 3x with RIP buffer and resuspended in 100 μl PBS. For isolation of immunoprecipitated protein, 20 μl of beads were transferred to another 1.5 ml tube and 6.7 μl of 4x Laemli buffer was added and the sample was incubated at 95°C for 5 minutes. The remaining beads were then treated with DNase 1 (Thermo Fisher Scientific) followed by Proteinase K (Thermo Fisher Scientific) to remove unwanted DNA and uncrosslinked protein. The tube was then placed on a magnetic stand and the supernatant containing the immunoprecipitated protein fraction was transferred to a new tube and frozen at -80°C. Trizol was added to the remaining 80 μl of beads and RNA was isolated as described above and resuspended in a final volume of 15 μl. For CLIP-RT-qPCR, 3 μl of immunoprecipitated eluate was used as the IP group and 1% of the input amount (5 μl) used in the input group for each RT reaction. RNA used in RIP-seq was first rRNA depleted followed by library construction using KAPA Stranded mRNA-Seq Kit (Illumina Platforms) (KR0960m Kapa Biosystems) with 10 cycles of PCR amplification and sequenced on an Illumina Hiseq2500.

### RNA-seq and data analysis

RNA from whole cells were polyA selected and RNAseq libraries were prepared using KAPA RNA HyperPrep Kit with RiboErase (HMR) (Illumina Platforms) (Kapa Biosystems), using 12 cycles of PCR amplification. Libraries were purified using AxyPrep Mag PCR Clean-up kit (Thermo Fisher Scientific). Sequencing was performed on an Illumina® HiSeq 2500 (Illumina) instrument. Nuclear or cytoplasmic fractions were rRNA-depleted and libraries were prepared similar to whole cell RNA. Data analysis of polyA RNA-seq of whole cells entailed mapping raw reads to a human transcriptome built with GENCODE v33 annotations 43 using STAR (version 2.7.2b) 44 and the expression of genes, including read counts and transcript per million (TPM), were calculated by RSEM (version 1.2.31) 45. Batch effect normalization was performed via the ComBat-seq function of sva (version 3.40) 46 and removed genes with read counts <5 in at least 50% of the samples prior to using DESeq2 (version 1.32.0) 47 to calculate the dispersion and fold change following YTHDC1 KO in MDA-MB-231 cells. Gene Ontology (GO) and Gene Set Enrichment Analysis (GSEA) analyses were performed by utilizing clusterProfiler4 (version 4.0.2) 48. For calculation of nuclear/cytoplasmic ratio of RNA following YTHDC1 KO, RNA-seq of nuclear and cytoplasmic fractions were analyzed with RUVSeq (version 1.26.0) 49 to remove any unwanted batch effects. The ratio of ratios test model was employed by DESeq2 to identify potential genes that are differentially retained in the nucleus in the YTHDC1 KO group. A transcript was considered as nuclear retained when it has a nuclear to cytoplasmic log_2_ fold change of 0.5 or higher when comparing sgB vs sgNS.

### RIP-seq analysis

Raw reads from RIP-seq were mapped to the transcriptome and quantified using the same data processing procedures described for RNA-seq above. To identify YTHDC1 target transcripts, we kept transcripts with read counts ≥ 5 in at least 50% of the samples, performed batch effect correction using RUVSeq, and calculated the dispersion and fold change of transcripts upon YTHDC1 knockdown in MDA-MB-231 cells by DESeq2 with shrinkage estimators. The transcripts with adjusted *P*-value ≤ 0.1 and fold change ≥ 2 were recognized as YTHDC1 bound targets.

### m^6^A-seq analysis

Raw reads from m^6^A-seq were mapped to the human transcriptome annotations (GENCODE v33) using STAR with the parameters -outFilterMismatchNoverReadLmax 0.04 and --outFilterMultimapNmax 20. Potential m^6^A peaks were identified using exomePeak (version 2.16) 50 with default parameters. Significant m^6^A peaks were defined as having a fold enrichment ≥ 1 and FDR ≤ 0.05.

### RT-qPCR for gene expression analysis

QuantiTect Reverse Transcription Kit (Qiagen) was used to synthesize cDNA for RT-qPCR, RIP-RT-qPCR and m^6^A-RT-qPCR using manufacturers protocols. For RT-qPCR, 500 ng of total RNA was used for each reaction. The cDNA samples were diluted 1:10 using nuclease-free water before qPCR analysis. For each qPCR reaction, 5 μl of Applied Biosystems PowerUp SYBR Green Master Mix (Thermo Fisher Scientific), 3 μl nuclease-free water, 1 μl mix of forward and reverse primers (5 μM), and 1 μl of cDNA was used in a 384-well format. The qPCR was run on a QuantStudio 12K Flex Real-Time PCR System (Thermo Fisher Scientific) using the standard cycling mode. β-actin was used as a loading control for gene expression analysis. A list of qPCR primers used is provided in Table S2.

### Immunofluorescence imaging

Cells seeded in 24-well plates were fixed with 4% formaldehyde at room temperature for 15 minutes and then permeabilized with 1% Triton-X100 in PBS for 15 minutes at room temperature. Wells were blocked by incubating with 10% FBS in PBS for one hour at room temperature and cells were stained with mouse anti-vimentin (1:200, MA511883, Thermo Fisher Scientific) overnight at 4°C. The cells were washed with PBS and stained with Alexa Fluor 488 goat anti-mouse secondary antibody (1:500, A11001, Thermo Fisher Scientific). After three PBS washes, cells were stained with DAPI and imaged on an Olympus BX61 imaging system. Cell aspect ratio was calculated using ImageJ. The threshold function was used on vimentin images to convert them to a binary image containing the outline of cells. Aspect ratio was determined using the analyze particles function, which was calculated by dividing by the length by the width of a cell.

### Fluorescence *in situ* hybridization

Custom Stellaris® FISH Probes were designed against the SMAD3 coding sequence by utilizing the Stellaris® RNA FISH Probe Designer (Biosearch Technologies, Inc.) available online at www.biosearchtech.com/stellarisdesigner (version 4.2). Samples were hybridized with the SMAD3 Stellaris RNA FISH Probe set labeled with Quasar® 570 dye (Biosearch Technologies, Inc.), following the manufacturer's instructions. Briefly, cells were fixed in 4% formaldehyde, permeabilized with 70% ethanol and incubated with probe overnight. The samples were mounted onto slides with Fluoroshield® medium containing DAPI (F6057, Sigma-Aldrich) and imaged using a Carl Zeiss LSM880 confocal laser scanning microscope.

### Dual luciferase assay

HEK293T cells were transfected with firefly luciferase fused to either WT or mutant *SMAD3* 3'UTR plasmids and a renilla luciferase plasmid using Effectene Transfection Reagent (Qiagen) according to the manufacturer's protocol. Two days later, cells were then transduced with lentivirus carrying *YTHDC1* shRNA as described above.

Three days after lentiviral transduction, cells were trypsinized and the Dual-Luciferase Reporter Assay System (Promega) was used for detection according to manufacturer's instructions. Briefly, lysate from 10,000 cells was pipetted into each well of a 96-well white bottom plate. Then, firefly luciferase substrate was added, and the signal was quantified on a GloMax® Navigator Microplate Luminometer (Promega). Lastly, a solution that quenches firefly luciferase activity and contains a substrate for renilla luciferase was added and the signal was quantified again. The firefly luciferase signal was normalized using the renilla luciferase signal.

### Statistical analyses

Statistical analyses of experimental data were calculated with Prism Version 8 (GraphPad Software). The log-rank test was used to determine significance for survival plots. For comparison between two samples, the Student's *t*-test was employed. One-way ANOVA with Dunnett's test was used for comparisons between more than two samples. For comparison of growth curves and primary tumor sizes, Two-way ANOVA with Tukey's multiple comparisons test was used. *P*-values less than 0.05 were considered significant.

### Data availability

The data generated in this study are publicly available in Gene Expression Omnibus (GEO) at GSE193156.

## Supplementary Material

Supplementary figures and tables.

## Abbreviations

ALKBH5: AlkB Homolog 5, RNA Demethylase
AML: Acute myeloid leukemia
CK18: Cytokeratin 18
CPTAC: Clinical Proteomic Tumor Analysis Consortium
CRISPR: clustered regularly interspaced short palindromic repeats
EMT: Epithelial to mesenchymal transition
EPHA1: EPH Receptor A1
FTO: FTO Alpha-Ketoglutarate Dependent Dioxygenase
GO: Gene ontology
GSEA: Gene set enrichment analysis
HPRT1: Hypoxanthine Phosphoribosyltransferase 1
IL11: Interleukin 11
m^6^A: *N^6^*-methyladenosine
METTL3: Methyltransferase 3, N6-Adenosine-Methyltransferase Complex Catalytic Subunit
METTL14: Methyltransferase 14, N6-Adenosine-Methyltransferase Subunit
MOI: Multiplicity of infection
KO: knockout
KD: knockdown
PCA: Principal component analysis
SMAD3: SMAD Family Member 3
SNAI1: Snail Family Transcriptional Repressor 1
SRSF3: Serine and Arginine Rich Splicing Factor 3
TCGA: The Cancer Genome Atlas
TNBC: triple negative breast cancer
TGF-β: Transforming Growth Factor Beta
TGFBR1: Transforming Growth Factor Beta Receptor 1
WT: Wild type
YTHDC1: YTH Domain Containing 1
YTHDF2: YTH *N^6^*-Methyladenosine RNA Binding Protein 2
