# Supplementary material for: RNA N6-methyladenosine reader YTHDC1 is essential for TGF-beta-mediated metastasis of triple negative breast cancer
Source: Theranostics. 2022 Jul 18;12(13):5727–43. doi: 10.7150/thno.71872 (PMC9373808; doi:10.7150/thno.71872)
Supplement: Supplementary file 1 — Supplementary figures and tables. [file thnov12p5727s1.pdf]

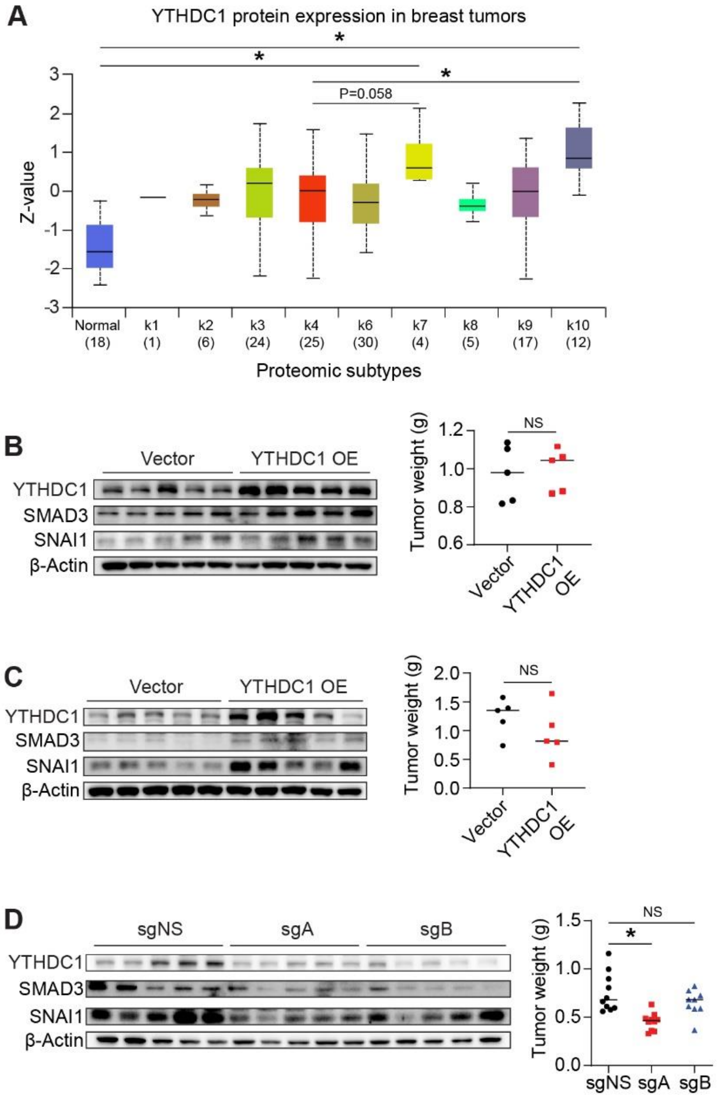

Figure S1. YTHDC1 protein expression in primary tumor samples and tumor weights. **A)** Box-whisker plot of YTHDC1 protein levels in breast tumors from different proteomic profile clusters. Proteomic profiles defined as: k1, Over-expression of proteasome complex proteins, glycolysis proteins, and pentose phosphate pathway proteins; k2, Adaptive immune system-related; k3, Innate immune system-related; k4, basal-like breast cancer; k6, Stromal-related, over-expression of matrix metallopeptidases; k7, Stromal-related, over-expression of collagen VI proteins; Wnt and Notch pathway signatures; k8, Over-expression of Golgi apparatus-related

proteins; k9, Found in clear cell renal cell carcinoma cases only; and k10, Over-expression of endoplasmic reticulum-related proteins. Numbers in parentheses indicate sample size. *t-test.* *$P < 0.05$, NS = not significant. **B)** Western Blot for primary tumors from MDA-MB-231 YTHDC1 OE mice (left) and primary tumor weight comparison (right). n = 5/group. *t*-test, NS = not significant. **C)** Western Blot for primary tumors from SUM159 YTHDC1 OE mice (left) and primary tumor weight comparison (right). n = 5/group. *t*-test, NS = not significant. **D)** Western Blot for primary tumors from MDA-MB-231 YTHDC1 KO mice (left) and primary tumor weight comparison (right). n = 5/group for Western Blot and 10/group for tumor weight. One-way ANOVA compared to sgNS, *$P < 0.05$, NS = not significant.

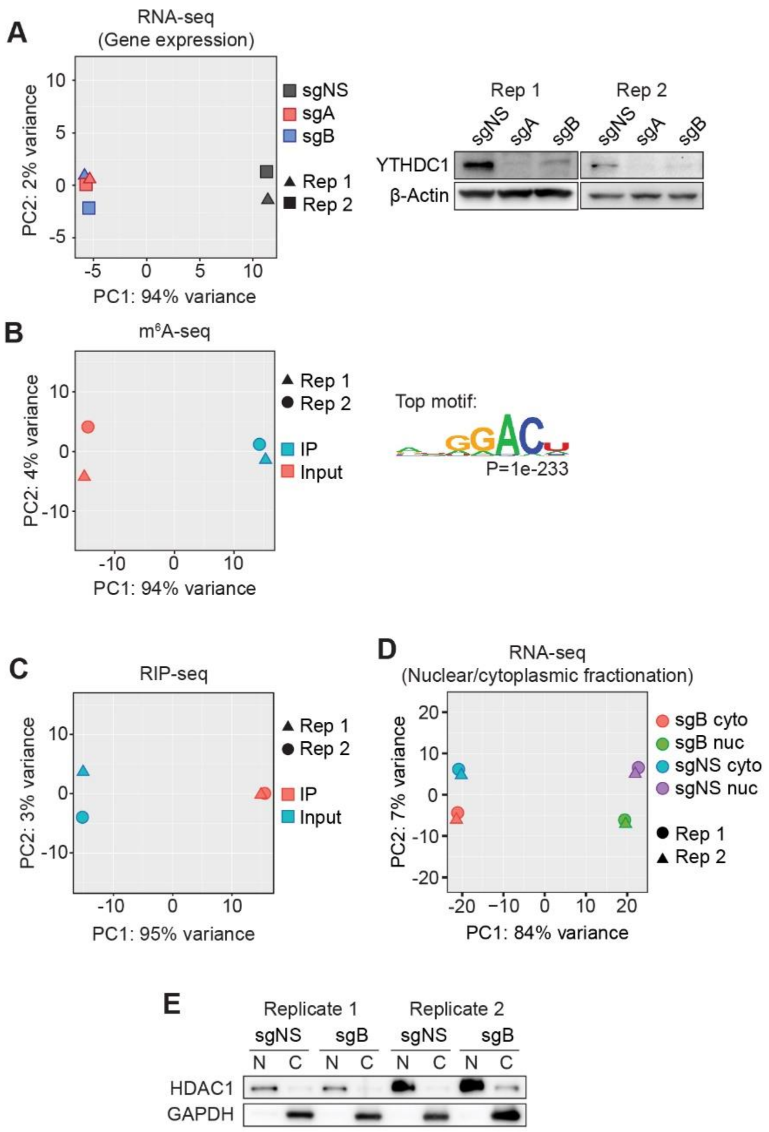

Figure S2. Reproducibility of sequencing data and verification of nuclear/cytoplasmic fractionation of MDA-MB-231 YTHDC1 KO cells by Western Blot. Two biological replicates were done for each experiment. **A)** PCA of poly(A) RNA-seq data from MDA-MB-231 YTHDC1 KO cells used to determine gene expression and Western Blot showing YTHDC1 KO efficiency. **B)** PCA of m^6A-seq data from MDA-MB-231 cells showing m^6A-immunoprecipitated (IP) and input groups and the most highly enriched motif from m^6A peaks. **C)** PCA of RIP-seq data from MDA-MB-231 cells overexpressing YTHDC1 showing Flag-

immunoprecipitated (IP) and input groups. **D)** PCA of RNA-seq data from nuclear and cytoplasmic fractions of MDA-MB-231 YTHDC1 KO cells (sgB) and control cells (sgNS). **E)** Western Blot verifying nuclear/cytoplasmic fractionation of MDA-MB-231 YTHDC1 KO and control cells used in nuclear/cytoplasmic RNA-seq. HDAC1 was used as a nuclear marker and GAPDH was used as a cytoplasmic marker.

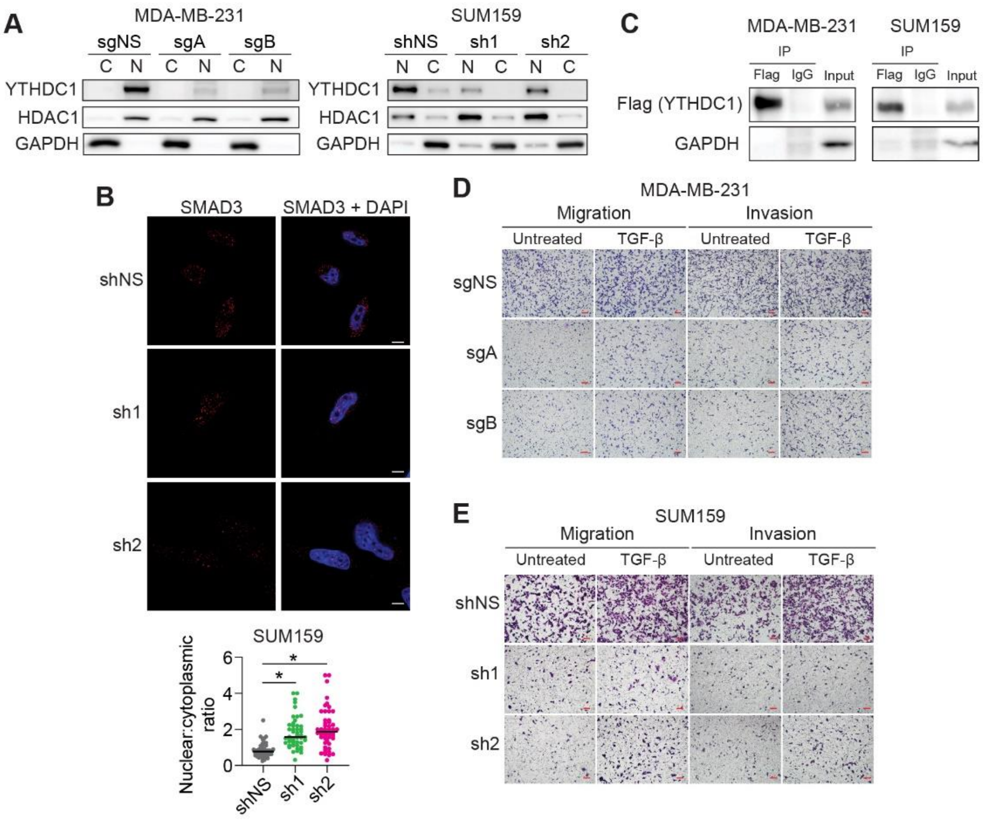

Figure S3. Western Blots for RIP-RT-qPCR and nuclear/cytoplasmic fractionation experiments and SMAD3 FISH. **A)** Nuclear/cytoplasmic fractionation of MDA-MB-231 YTHDC1 KO cells and SUM159 YTHDC1 KD cells. **B)** Representative images and quantification of SMAD3 mRNA localization by FISH following YTHDC1 KD in SUM159 cells. Scale bar: 10 μm. Each point on the graph represents a single cell. One-way ANOVA compared to sgNS. n = 50/group. **C)** Western Blots showing immunoprecipitation efficiency of Flag-YTHDC1 in MDA-MB-231 and SUM159 cells. **D-E)** Transwell migration and invasion images for **(D)** MDA-MB-231 YTHDC1 KO or **(E)** SUM159 YTHDC1 KD cells treated with or without TGF-β. Scale bar: 100 μm.

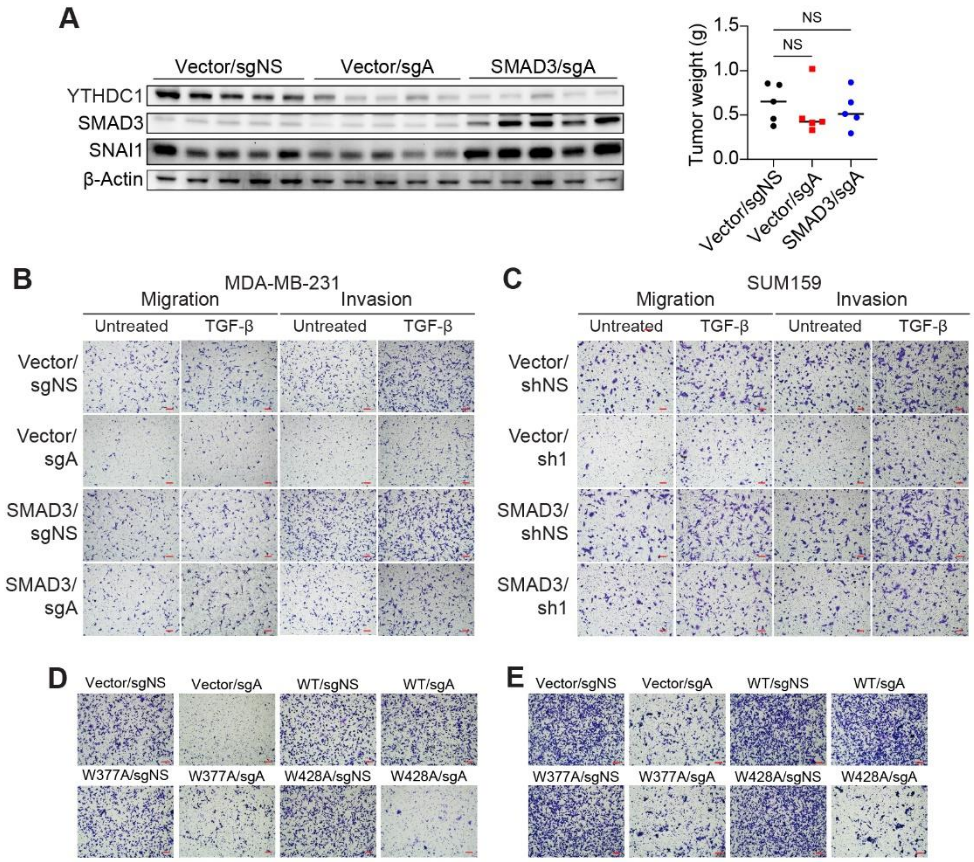

Figure S4. SMAD3 *in vivo* rescue experiment and Transwell migration and invasion images. **A)** Western Blot for primary tumors from MDA-MB-231 YTHDC1 KO SMAD3 rescue mice (left) and weight of primary tumor comparison (right). n = 5/group. One-way ANOVA, NS = not significant. **B-C)** Images from Transwell migration and invasion assay of **(B)** MDA-MB-231 YTHDC1 KO or **(C)** SUM159 YTHDC1 KD cells overexpressing SMAD3 treated with TGF-β. **D-E)** Transwell migration **(D)** and invasion **(E)** images of YTHDC1 KO or control MDA-MB-231 cells overexpressing different YTHDC1 mutants. Scale bar: 100 μm.

Table S1. List of plasmids.

Plasmid name	Source	Notes
psPAX2	Addgene 12260	A gift from Didier Trono.
PMD2.G	Addgene 12259	A gift from Didier Trono.
lentiCas9-Blast	Addgene 52962	A gift from Feng Zhang.
pCDH-CMV-3xflag-YTHDC1-Puro	This paper	Used for MCF10A YTHDC1 overexpression experiment, animal studies, RNA-stability experiments, CLIP-RT-qPCR and RIP-seq.
pCDH-CMV-3xflag-YTHDC1 WT-mutA-Puro	This paper	Synonymous mutations at the sgA site. Used in rescue experiments.
pCDH-CMV-3xflag-YTHDC1 W377A-mutA-Puro	This paper	Synonymous mutations at the sgA site. Used in rescue experiments.
pCDH-CMV-3xflag-YTHDC1 W428A-mutA-Puro	This paper	Synonymous mutations at the sgA site. Used in rescue experiments.
PLX-CMV-myc-SMAD3-Blast	This paper	
pGL3-basic-firefly luc-WT-SMAD3 3'UTR	This paper	WT SMAD3 3'UTR cloned into pGL3-basic-firefly luciferase vector (E1751, Promega).
pGL3-basic-firefly luc-mut-SMAD3 3'UTR	This paper	Mutant SMAD3 3'UTR cloned into pGL3-basic-firefly luciferase vector (E1751, Promega).
PRL-TK (renilla luciferase)	Promega E2241	

Table S2. List of oligonucleotides used for RT-qPCR.

Name	Sequence
Human ACTB F	CACTCTTCCAGCCTTCCTTC
Human ACTB R	GTACAGGTCTTTGCGGATGT
Human SMAD3 F	AACTCAAGAAGACGGGGCAG
Human SMAD3 R	CTGGGGATGGTGATGCACTT
Human SMAD3 m^6A-RT-qPCR F	CTGTTGCAACTCGGCTGTTC
Human SMAD3 m^6A-RT-qPCR R	AGGCTGGCCGAATAGTGAAG
Human HPRT1 F	TTGCTTTCCTTGGTCAGGCA
Human HPRT1 R	ATCCAACACTTCGTGGGGTC
Human SNAI1 F	TGCCCTCAAGATGCACATCCGA
Human SNAI1 R	GGGACAGGAGAAGGGCTTCTC
Human Fibronectin F	ACAACACCGAGGTGACTGAGAC
Human Fibronectin R	GGACACAACGATGCTTCCTGAG

Human IL11 F	TTCAGTACTGGGGGCGAAAC
Human IL11 R	AATAAGGCACAGATGCCCCC
Human CDH1 F	GCCTCCTGAAAAGAGAGTGGAAG
Human CDH1 R	TGGCAGTGTCTCTCCAAATCCG
Human CLDN7 F	TAGCTTGCTCCTGGTATGGC
Human CLDN7 R	TGGCAGGGCCAAACTCATAC
Firefly luciferase F	ATGGAAGACGCCAAAAACATAAAG
Firefly luciferase R	GCGGAACTCCCAAGCTTATCG
